# Supplementary material for: Main Concept, Sequencing, and Story Grammar Analyses of Cinderella Narratives in a Large Sample of Persons with Aphasia
Source: Brain Sci. 2021 Jan 15;11(1):110. doi: 10.3390/brainsci11010110 (PMC7830981; doi:10.3390/brainsci11010110)
Supplement: Supplementary file 1 [file brainsci-11-00110-s001.pdf]

Supplemental Files

Table S1. Goodness of fit statistics for each distribution and link tested for all MSSG variables with PNBIs.

Distribution + Link	Pearson χ^2/df	Log Likelihood	Akaike Information Criteria (AIC)
**MC Composite**			
Normal + Identity	225.783	−452	914
Tweedie + Identity	0.516	−461	933
Poisson + Identity	3.897	−549	1106
**Sequencing**			
Normal + Identity	234.956	−454	918
Tweedie + Identity	0.516	−464	938
Poisson + Identity	3.949	−554	1116
**MC + Sequencing**			
Normal + Identity	914.142	−529	1068
Tweedie + Identity	0.724	−538	1087
Poisson + Identity	7.787	−813	1634
**Essential Story Grammar**			
Normal + Identity	27.32	−335	681
Tweedie + Identity	0.299	−344	698
Poisson + Identity	1.340	−343	695
**Total Episodic Components**			
Normal + Identity	4.566	−237	485
Tweedie + Identity	0.124	−247	504
Poisson + Identity	0.412	−259	526
**Episodic Complexity**			
Normal + Identity	0.857	−145	301
Tweedie + Identity	0.222	−191	391
Multinomial + Cumulative logit	0.915	−123	261

Table S2. Goodness of fit statistics for each distribution and link tested for all MSSG variables with PWAs.

Distribution + Link	Pearson χ^2/df	Log Likelihood	Akaike Information Criteria (AIC)
**MC Composite**			
Normal + Identity	325.181	−1368	2747
Gamma + Identity	0.597	−1182	2375
Tweedie + Identity	3.431	−1278	2566
Poisson + Identity	15.399	−3150	6308
**Sequencing**			
Normal + Identity	395.887	−1400	2810
Gamma + Identity	0.476	−1228	2467
Tweedie + Identity	3.139	−1328	2667
Poisson + Identity	15.568	−3332	6672
**MC + Sequencing**			
Normal + Identity	1424.485	−1603	3217
Gamma + Identity	0.522	−1407	2824
Tweedie + Identity	4.566	−1504	3019
Poisson + Identity	30.562	−5847	11703
**Essential Story Grammar**			
Normal + Identity	46.664	−1060	2130
Gamma + Identity	0.466	−921	1852
Tweedie + Identity	1.813	−1021	2052
Poisson + Identity	5.291	−1453	2915
**Total Episodic Components**			
Normal + Identity	15.639	−886	1782
Gamma + Identity	0.366	−729	1469
Tweedie + Identity	1.371	−873	1757
Poisson + Identity	3.054	−1032	2073
**Episodic Complexity**			
Normal + Identity	2.408	−588	1187
Gamma + Identity	0.212	−323	657
Tweedie + Identity	1.198	−595	1201
Poisson + Identity	1.487	−563	1135
Multinomial + Cumulative logit	0.997	−499	1014
